# Resistance profile and biofilm production of microorganisms isolated in operating rooms

**DOI:** 10.1590/0034-7167-2025-0189

**Published:** 2026-09-28

**Authors:** Vanessa Oliveira Lopes de Moura, Tainá Francisca Cardozo de Oliveira, Eli Júnior Pereira Rodrigues, Vanessa Bridi, Stéfanne Rodrigues Rezende Ferreira, Ludimila Paula Vaz Cardoso, Berendina Elsina Bouwman, Hanstter Hallison Alves Rezende

**Affiliations:** I Universidade Federal de Jataí. Jataí, Goiás, Brazil

**Keywords:** Surgicenters, Cross Infection, Microbial Drug Resistance, Staphylococcus aureus, Biofilms., Centros Quirúrgicos, Infección Hospitalaria, Farmacorresistencia Microbiana, Staphylococcus aureus, Biopelículas.

## Abstract

**Objectives:**

to evaluate the resistance profile and biofilm formation in isolates from operating rooms in two hospitals in the southwestern region of Goiás.

**Methods:**

an experimental laboratory study was conducted, with samples collected between March and August 2023.

**Results:**

of the 50 samples collected, 104 bacteria were isolated, 81.7% (85/104) gram-positive and 18.3% (19/104) gram-negative. Of these, 55.3% (47/85) of the Staphylococcus genus were classified as multidrug-resistant and methicillin-resistant, and 15.7% (3/19) of the Gram-negative bacteria were classified as multidrug-resistant. 66.34% (69/104) were biofilm producers, with 79.7% (55/69) being Gram-positive and 20.3% (14/69) Gram-negative.

**Conclusions:**

this finding is of great concern, as these microorganisms in hospital environments pose a high risk for surgical site infections that are difficult to treat due to multidrug-resistant strains.

## RESUMEN

**Objetivos:**

evaluar el perfil de resistencia y formación de biopelículas en aislados de quirófanos de dos hospitales de la región suroeste de Goiás.

**Métodos:**

estudio experimental de laboratorio, en el que se realizaron recolecciones durante los meses de marzo a agosto de 2023.

**Resultados:**

de las 50 muestras recogidas, se aislaron 104 bacterias, 81,7 % (85/104) grampositivas y 18,3 % (19/104) gram-negativas. De estas, el 55,3 % (47/85) del género Staphylococcus se clasificaron como multirresistentes y resistentes a la meticilina, y el 15,7 % (3/19) de las gram-negativas como multirresistentes. El 66,34 % (69/104) eran productoras de biofilm, siendo el 79,7 % (55/69) gram-positivas y el 20,3 % (14/69) gram-negativas.

**Conclusiones:**

este hallazgo es muy preocupante, ya que estos microorganismos en entornos hospitalarios presentan un alto riesgo de infecciones del sitio quirúrgico difíciles de tratar debido a las cepas multirresistentes.

## INTRODUCTION

The hospital environment is a large reservoir of virulent microorganisms that can cause Healthcare-Associated Infections (HAIs) in patients, especially in cases of prolonged hospitalizations, invasive and surgical procedures, and also due to exogenous factors related to the environment, materials, and equipment. In Brazil, concern about healthcare-associated infections (HAIs) has been growing since the 1990s, and they currently represent the fourth leading cause of mortality in Intensive Care Units (ICUs)^(1,2)^.

Data from the National Biosafety Agency (ANBIO) indicate that 80% of hospitals do not adequately control HAIs. Furthermore, data from the National Regulatory Agency for Private Health Insurance and Plans (ANS) indicates that more than 45,000 Brazilians die annually as a result of these diseases, and the World Health Organization (WHO) estimates this number to be even higher, reaching 100,000 cases per year^(3-5)^.

The interaction of different microorganisms in the same environment, especially in hospitals, favors the development of some mechanism of resistance to antimicrobials^(6,7)^, which occurs due to various mutations, hindering the treatment of bacterial infections and potentially leading to unfavorable clinical outcomes, and even patient death^(6,8,9)^.

Another aggravating factor of hospital microorganisms is their ability to produce biofilms, which consist of a layer of extracellular polymeric substance (EPS), composed of polysaccharides, extracellular DNA, proteins, lipids, surfactants, flagella, and pili, thus providing protection to the microorganisms present there, as they are highly resistant to antimicrobial agents and environmental disinfectants^(10,11)^.

Inside biofilms, these microorganisms have the ability to interact/communicate with each other, a process called *Quorum sensing* (QS). This factor facilitates the transfer of multidrug-resistant plasmids, favoring the development of persistent infectious diseases. According to the National Institutes of Health in the United States, approximately 80% of all infections worldwide are associated with the presence of biofilms^(10,11)^.

Some hospital environments are more susceptible to the presence and growth of microorganisms, and among these is the operating room, as it is a place where many patients and professionals pass through, where several types of invasive procedures are performed, in addition to artificial climate control and the use of materials and equipment^(12,13)^. These infections can be classified as exogenous when they originate from a source external to the patient, or endogenous when they originate from the patient themselves. Exogenous transmission is primarily through direct contact, but it can also occur indirectly through contaminated equipment, instruments, and surfaces, or even through improperly processed implant materials^(14-17)^.

Due to all these related factors, HAIs, particularly surgical site infections (SSIs), are a significant concern for patient safety and can occur in both major surgeries and minor procedures^(18)^.

## OBJECTIVES

To identify, evaluate the antimicrobial resistance profile, and verify the biofilm formation of microorganisms isolated from surfaces in operating rooms.

## METHODS

### Study type or design

Ethical review and approval were waived for this study, as it is an experimental laboratory study of surface sample collection, based on *Revised Standards for Quality Improvement Reporting Excellence* (SQUIRE 2.0)^®(19)^.

### Local

The study was conducted in the operating room of two healthcare facilities located in the Southwest of Goiás. One of medium complexity and another of high complexity, three and four operating rooms, providing services in various areas such as internal medicine, pediatrics, obstetrics, general surgery, orthopedics, urology, ophthalmology, otolaryngology, among others.

The laboratory analyses were developed in the Bacteriology and Mycology Laboratory of the Biomedicine course at the Federal University of Jataí - GO (LBM-UFJ).

### Period and collection of samples

The collections were carried out between the months of March to August 2023. To identify and evaluate the antimicrobial resistance profile and verify biofilm formation, samples were collected using sterile *Swabs* moistened with a 0.9% saline solution, the samples were rubbed over the entire surface of the selected objects, which were: computer keyboards and mouses, suction machine cords, electrosurgical unit cords, electrosurgical unit foot pedal, cardiac monitor lead connectors, and blood pressure monitor cuffs, obtaining a total of 50 samples.

After collection, swabs were placed in previously identified test tubes containing *Brain Heart Infusion* (BHI) (HIMEDIA^®^) broth, packaged in a thermal box, and transported to LBM-UFJ, where they remained incubated for 24 h in a Biochemical Oxygen Demand (BOD) incubator at a temperature of 35°C-37°C.

### Microbiological analyses

Bacterial growth was determined based on the BHI (HIMEDIA^®^) broth cloudiness. Samples showing growth were streaked onto Blood Agar - AS (KASVI^®^) and incubated for 24 hours in a BOD incubator at a temperature of 35°C-37°C. After the incubation period, it was observed whether there was growth, as well as the number of distinct colonies on each plate. The morphologically different colonies were re-inoculated (secondary isolation) to achieve the purity of each colony, allowing the performance of further identification tests. The previously isolated samples (observing hemolysis patterns and Gram staining) were subjected to traditional biochemical tests for identification^(20)^.

### Analysis of resistance and biofilm production

The Kirby-Bauer disk diffusion method was used to perform the antimicrobial susceptibility test^(21)^, depositing antimicrobial discs in a Petri dish (140x15mm) containing Mueller Hinton agar (HIMEDIA^®^) previously inoculated with an isolated bacterial strain of approximately 1.5 x 10^8^ CFU/ml (0.5 of the scale of *McFarland*), incubated at 35-37ºC for 24h. The results were interpreted by comparing the diameter of the inhibition halo with the classifications of the cut-off points established by the *Brazilian Committee on Antimicrobial Susceptibility Testing* (BrCAST) and in this way they were classified as sensitive, intermediate, or resistant^(21)^.

The resistance profile of the *Staphylococcus* group to oxacillin (with the resistant ones being classified as MRSA - methicillin-resistant *Staphylococcus aureus*, MRSE - methicillin-resistant *Staphylococcus epidermidis*, and MRSS- methicillin-resistant *Staphylococcus saprophyticus*), of the *Enterococcus* sp. group to vancomycin (with the resistant ones being classified as VRE - vancomycin-resistant *Enterococcus*) and Enterobacteriaceae resistant to imipenem (with the resistant ones classified as: possible KPC)^(21)^.

For the classification of the multiresistance profile, the criteria proposed by Magiorakos *et al.* ^(22)^ were adopted, where isolates that did not show resistance or showed up to two classes were classified as Not MDR (*Not multidrug resistant*); isolates that showed resistance to at least one representative in three or more classes of antimicrobials were classified as MDR (*multidrug resistance*); and the isolates showing resistance to all agents from all classes were classified as PDR (*pan drug resistance*)^(22)^.

For conducting tests of biofilm production, the isolates were thawed and an aliquot was transferred to a test tube containing BHI broth. After 24 hours of incubation, the biofilm was mounted in 96-well plates containing 198 μL of Tryptic Soy Broth enriched medium - TSB (HIMEDIA^®^) and 2 μL of BHI + Strain, this being incubated at 35°C-37°C in a BOD incubator for 48h. Subsequently, the steps described by Stepanovic *et al.* ^(23)^ were followed, which included the plate reading phase: plate washing, fixation and staining, and finally, measurement of results using a microtiter plate reader (KASVI^®^). The interpretation was expressed through the cutoff point *(Cut off* - ODc) described in Chart 1.

**Chart 1:** Interpretation of biofilm production

Classification	Analysis between OD and ODc^*^
Non-biofilm producer	OD ≤ ODc
Weak producer (+ or 1)	ODc < OD ≤ 2X ODc
Moderate producer (++ or 2)	ODc < OD ≤ 4X ODc
Strong producer (+++ or 3)	4X ODc < OD

*Source: Stepanovic et al.^(23)^.*

## RESULTS

The results obtained from the microbiological analysis of samples collected in two hospitals provide a comprehensive view of the presence of microorganisms in these facilities. Of the 50 samples analyzed, all showed growth of at least one type of microorganism, totaling 104 isolated bacteria. Characterization of the bacteria based on Gram staining revealed a predominance of Gram-positive bacteria, representing 81.7% (85/104) of the total, compared to Gram-negative bacteria, which constituted 18.3% (19/104).

Figure 1 provides a representation of the bacterial species identified (*Staphylococcus* sp*. (4), Staphylococcus epidermidis (20), Staphylococcus aureus (38), Staphylococcus saprophyticus (7), coagulase-negative Staphylococcus (11), Streptococcus viridans (1), Enterococcus* sp*. (4), Proteus mirabilis (3), Escherichia coli (7), Shigella* sp*. (3) and Acinetobacter* sp. *(5).*

**Figure 1:**
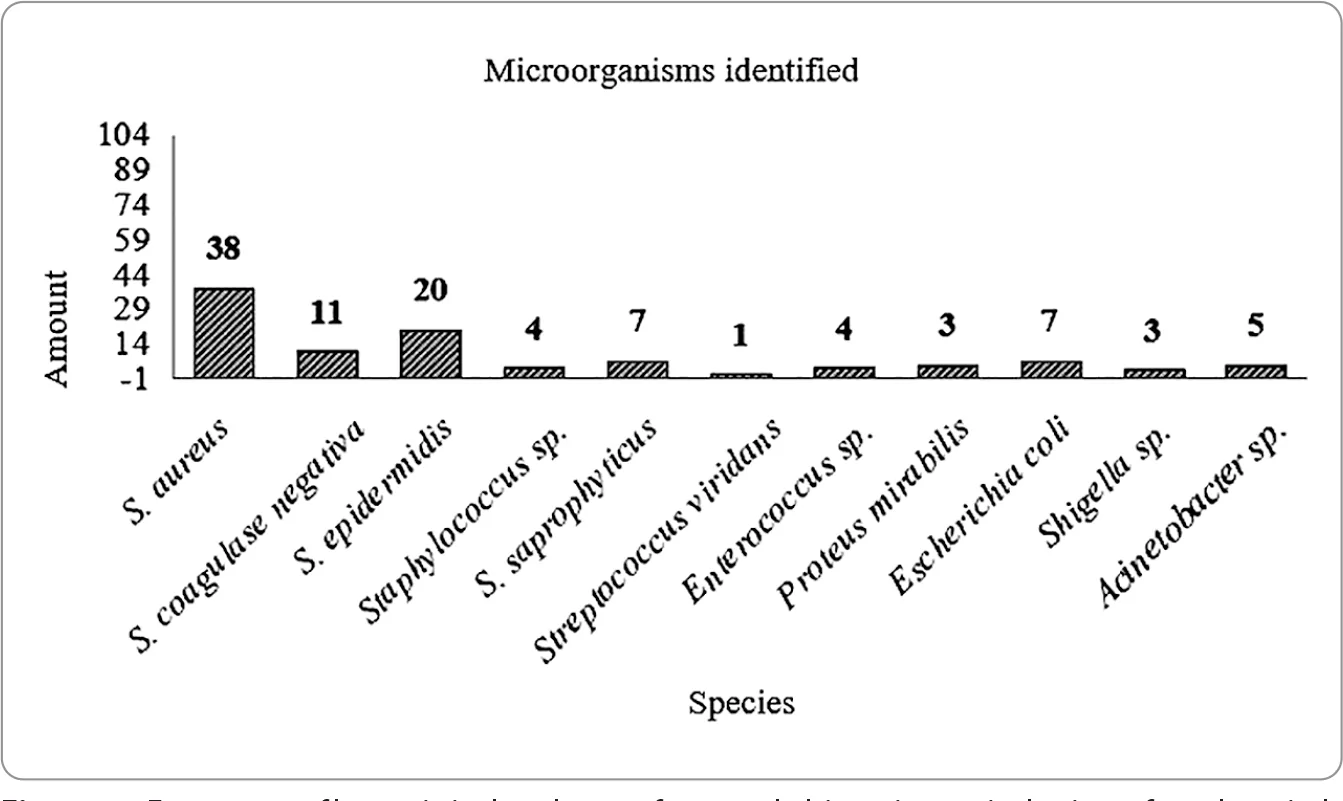
Frequency of bacteria isolated on surfaces and objects in surgical suites of two hospitals in southwestern Goiás

Regarding resistance mechanisms, in the present study it was observed that 57.7% (60/104) of the isolates showed resistance to antimicrobials and were classified as MDR according to Magioarakos *et al.* ^(12)^ criteria. Moving on to the evaluation of biofilm formation capacity, it was observed that 66.3% (69/104) of the isolates have this characteristic, with 89.8% (62/69) being weak producers, 5.8% (4/69) moderate producers, and 4.4% (3/69) strong producers, as shown in Chart 2.

**Chart 2:** Sample origin, resistance profile, and biofilm production capacity

Isolate	Surface/Object	Resistance to classes of antimicrobials	Biofilm Production	Isolate	Surface/Object	Resistance to classes of antimicrobials	Biofilm Production
1	Keyboard	MDR/MRSA	Non-producer	20	Electrosurgical unit cord	MDR/MRSE	Non-producer
2	Mouse	MDR/MRSA	Non-producer	21	Scalpel pedal	Not MDR	Non-producer
3	Mouse	MDR/MRSA	Non-producer	22	Scalpel pedal	Not MDR	Weak producer
4	Keyboard	MDR/MRSA	Non-producer	23	Scalpel pedal	Not MDR	Non-producer
5	Keyboard	MDR	Non-producer	24	BP device	Not MDR	Non-producer
6	Mouse	MDR	Non-producer	25	Lead Connector	MDR/MRSE	Non-producer
7	Keyboard	MDR/MRSA	Non-producer	26	Lead Connector	Not MDR	Non-producer
8	Keyboard	MDR	Non-producer	27	Sucction machine cord	Not MDR	Non-producer
9	Mouse	Not MDR	Non-producer	28	Sucction machine cord	Not MDR	Non-producer
10	Keyboard	MDR	Weak producer	29	Electrosurgical unit cord	Not MDR	Weak producer
11	Mouse	MDR/MRSA	Non-producer	30	Electrosurgical unit cord	MDR/MRSE	Weak producer
12	BP cuff	Not MDR	Non-producer	31	Electrosurgical unit cord	MDR	Non-producer
13	BP cuff	Not MDR	Weak producer	32	Electric scalpel pedal	Not MDR	Non-producer
14	BP cuff	MDR/MRSE	Average producer	33	Scalpel pedal	MDR	Weak producer
15	Lead Connector	MDR/MRSA	Weak producer	34	Scalpel pedal	MDR/MRSE	Weak producer
16	Lead Connector	MDR/MRSE	Non-producer	35	BP cuff	MDR/MRSA	Weak producer
17	Lead Connector	MDR/MRSA	Non-producer	36	BP cuff	MDR/MRSE	Non-producer
18	Sucction machine cord	Not MDR	Weak producer	37	Lead Connector	MDR/MRSE	Weak producer
19	Sucction machine cord	Not MDR	Weak producer	38	Sucction machine cord	Not MDR	Weak producer
39	Sucction machine cord	Not MDR	Weak producer	60	Keyboard 3	Not MDR	Non-producer
40	Electric scalpel cord	Not MDR	Weak producer	61	Keyboard 3	Not MDR	Weak producer
41	Electric scalpel cord	MDR	Weak producer	62	Keyboard 3	MDR/MRSA	Weak producer
42	Scalpel pedal	Not MDR	Non-producer	63	Mouse 3	MDR	Strong producer
43	Scalpel pedal	Not MDR	Weak producer	64	Mouse 3	Not MDR	Weak producer
44	Keyboard 1	MDR	Weak producer	65	Keyboard 4	Not MDR	Moderate producer
45	Keyboard 1	Not MDR	Weak producer	66	Keyboard 4	MDR/MRSA	Weak producer
46	Keyboard 1	Not MDR	Weak producer	67	Mouse 4	MDR	Weak producer
47	Keyboard 2	Not MDR	Weak producer	68	Keyboard 5	MDR	Weak producer
48	Keyboard 2	Not MDR	Non-producer	69	Mouse 5	MDR	Moderate producer
49	Keyboard 2	MDR/MRSE	Weak producer	70	Keyboard 6	MDR/MRSA	Weak producer
50	Keyboard 2	MDR/MRSA	Weak producer	71	Mouse 6	MDR/MRSA	Weak producer
51	Mouse 1	MDR/MRSA	Weak producer	72	Mouse 6	MDR/MRSA	Weak producer
52	Mouse 1	Not MDR	Non-producer	73	BP device	MDR/MRSA	Weak producer
53	Mouse 1	Not MDR	Non-producer	74	BP device	MDR/MRSA	Non-producer
54	Mouse 2	Not MDR	Non-producer	75	BP device	MDR/KPC	Weak producer
55	Mouse 2	MDR	Weak producer	76	BP device	Not MDR	Weak producer
56	Mouse 3	MDR	Non-producer	77	Lead Connector	MDR/MRSE	Weak producer
57	Mouse 4	Not MDR	Weak producer	78	Lead Connector	MDR/KPC	Weak producer
58	Mouse 5	MDR/MRSE	Weak producer	79	Sucction machine cord	Not MDR	Non-producer
59	Keyboard 3	MDR/MRSE	Weak producer	80	Electric scalpel cord	Not MDR	Non-producer
81	Electric scalpel cord	Not MDR	Weak producer	93	Sucction machine cord	MDR/MRSA	Weak producer
82	Scalpel pedal	MDR/MRSA	Non-producer	94	Electric scalpel cord	MDR/MRSA	Weak producer
83	BP device	Not MDR	Weak producer	95	Electric scalpel cord	Not MDR	Weak producer
84	BP device	Not MDR	Moderate producer	96	Scalpel pedal	MDR/MRSA	Weak producer
85	Lead Connector	MDR/MRSA	Weak producer	97	Scalpel pedal	Not MDR	Weak producer
86	Sucction machine cord	MDR/MRSA	Weak producer	98	Scalpel pedal	Not MDR	Weak producer
87	Electric scalpel cord	MDR/MRSA	Weak producer	99	Scalpel pedal	Not MDR	Weak producer
88	Scalpel pedal	MDR/MRSA	Strong producer	100	Scalpel pedal	MDR/MRSA	Weak producer
89	Scalpel pedal	Not MDR	Weak producer	101	Monitor cable	MDR	Weak producer
90	BP device	MDR/MRSA	Weak producer	102	Monitor cable	Not MDR	Weak producer
91	BP device	MDR/MRSA	Weak producer	103	Monitor cable	MDR/MRSE	Weak producer
92	Lead Connector	MDR/MRSA	Strong producer	104	Scalpel pedal	Not MDR	Weak producer

*Not MDR - No drug resistance; MDR - multidrug resistance; MRSA - methicillin resistant Staphylococcus aureus; MRSE - methicillin resistant Staphylococcus epidermidis; MRSS - methicillin resistant Staphylococcus saprophyticus; BP - blood pressure; KPC - carbapenemase-producing Klebsiella group.*

## DISCUSSION

Among HAIs, SSI stands out as a major concern in the hospital setting, and they can be caused by a wide range of bacteria, both gram-negative and/or gram-positive ones^(17)^. In the operating room, when preventive measures are not implemented or are neglected, the risk of infection increases considerably, potentially causing temporary or permanent damage, and even death to patients^(24)^.

This study showed that microorganisms were isolated from all surfaces where samples were collected, and among these were species that cause HAIs, with a significant resistance profile to antimicrobials, in addition to the capacity for biofilm production. These findings corroborate a study conducted in a hospital in Belo Horizonte, with the same objective, pointing to significant data related to the incidence of microorganisms on surfaces and objects in an operating room, namely: Coagulase-negative *Acinetobacter baumannii, Enterobacter* sp., and *Staphylococcus,* bacteria that are potential causes of HAIs, particularly SSIs, given the presence of isolates with high antimicrobial resistance profiles^(25)^.

Another study^(13)^ evaluated the bacterial profile found on the surfaces and equipment of an operating room, and microorganisms were isolated from all of them, with a prevalence of gram-positive cocci, with *S. aureus* being the most frequent occurrence, corroborating the findings of this study, where all surfaces and objects tested showed bacterial growth, with a predominance of the genre *Staphylococcus,* and the prevalence of *S. aureus* being highlighted. This pathogen is part of the human skin microbiota, but it can cause various infections in hospital settings, such as respiratory, systemic, and surgical site infections.

Similar research^(26)^ also pointed to a higher prevalence of the species *S. aureus,* which is closely linked to the development of HAIs, primarily by methicillin-resistant strains (MRSA). In the present study, 77.8% (81/104) of the isolates are of the genus *Staphylococcus.* Of these, 58.02% (47/81) showed resistance to oxacillin, classified as MDR/MRSA, and 66.66% (54/81) are biofilm-producing strains. It should be noted that this species has the ability to produce biofilms, which hinders the immune response and pharmacological therapy^(17)^.

*Enterococcus* sp. consists of a genus of Gram-positive bacteria naturally found in the gastrointestinal tract; however, studies indicate the presence of species within this genus associated with hospital-acquired infections, which is particularly relevant when these bacteria develop vancomycin resistance (VRE) mechanisms, as they can cause opportunistic infections that spread easily in the hospital environment. Study conducted at a university hospital^(12)^ points to high prevalence rates for *Enterococcus* sp. in various sectors of the hospital, being the second most frequently found in the samples of surgical incisions secretion. This study, in turn, isolated 3.84% (0.5/104) of the samples, and all of these proved sensitive to vancomycin; however, 75% (03/04) of the isolates were classified as MDR, thus pointing to an imminent risk of developing SSI, given that these microorganisms are producers of surface polysaccharides (*enterococcal polysaccharide antigen*) essential for biofilm development, which is directly linked to antimicrobial resistance as well as pathogenesis^(27)^.

In the present study, 18.3% (19/104) of the bacteria isolated were gram-negative (*E. coli, Proteus mirabilis, Acinetobacter sp. and Shigella sp.)* species, where 21.05% (04/19) exhibited multidrug resistance (MDR) to the tested antimicrobials. Furthermore, this study also corroborates the findings of a previous research project^(28)^ that shows that superbacteria are one of the biggest challenges faced in the hospital environment, especially in SSIs.

Moreover, the biofilm production capacity was evaluated, as this is a characteristic associated with bacterial virulence and resistance. The presence of biofilm can contribute to the persistence of HAIs and hinder appropriate treatment^(29)^. The analysis revealed variations in biofilm production, with 66.34% (69/104) of the isolates classified as biofilm producers, 79.7% (55/69) as gram-positive bacteria and 20.3% (14/69) as gram-negative bacteria. The same was shown in a study^(30)^ conducted at a hospital in Pokhara, Nepal, which assessed the contamination of frequently touched objects, and found a higher prevalence of *S. aureus* in the 181 samples analyzed, with 36.3% being methicillin-resistant and 31.8% being biofilm producers.

### Study limitations

As the bacterial strains were being identified, the performance of several subcultures of the same sample was required so that the colony could be isolated to proceed with the identification flowchart. Therefore, a single sample required around 5 to 6 days for identification, due to the fact that a single plate exhibited polymicrobial growth.

### Contributions to the fields of nursing, health, or public policy

HAIs can be prevented and controlled through the implementation of infection prevention and control measures at all levels of healthcare. Preventing HAIs is the responsibility of healthcare facilities and all staff members. Everyone must work together to reduce the risk of infection for both patients and healthcare workers themselves^(31)^. The association between antimicrobial resistance and biofilm production highlights the need for the implementation of integrated infection control strategies, emphasizing the fundamental role of healthcare professionals, the early identification of bacteria, and the implementation of appropriate preventive measures essential to mitigating the risks of hospital-acquired infections associated with these microorganisms^(32,33)^.

## CONCLUSIONS

The results presented provide valuable insights for hospital health management, highlighting the importance of continuous surveillance, infection control practices, and the development of personalized therapeutic strategies to address microbiological diversity and antimicrobial resistance in hospital settings. Given these findings, it is necessary for staff working in the Operating Room (OR) to adopt improved cleaning and disinfection measures for surfaces and objects aiming to reduce the risk of cross-infections.

## Data Availability

The research data are available within the article.
