# Supplementary material for: Resistance profile and biofilm production of microorganisms isolated in operating rooms
Source: Rev Bras Enferm. 2026 Sep 28;79:e20250189. doi: 10.1590/0034-7167-2025-0189 (PMC13623299; doi:10.1590/0034-7167-2025-0189)
Supplement: Translation

# Translation: Perfil de resistência e produção de biofilmes de microrganismos isolados em centros cirúrgicos

**DOI:** 10.1590/0034-7167-2025-0189pt

**Authors:** Vanessa Oliveira Lopes de Moura, Tainá Francisca Cardozo de Oliveira, Eli Júnior Pereira Rodrigues, Vanessa Bridi, Stéfanne Rodrigues Rezende Ferreira, Ludimila Paula Vaz Cardoso, Berendina Elsina Bouwman, Hanstter Hallison Alves Rezende

**Affiliations:** I Universidade Federal de Jataí. Jataí, Goiás, Brasil

**Keywords:** Centros Cirúrgicos, Infecção Hospitalar, Resistência Microbiana a Medicamentos, Staphylococcus aureus, Biofilmes.

## Abstract

**Objetivos:**

avaliar o perfil de resistência e formação de biofilme em isolados de centros cirúrgicos de 2 hospitais da região sudoeste de Goiás.

**Métodos:**

estudo de caráter experimental laboratorial, onde foram realizadas coletas durante os meses de março a agosto de 2023.

**Resultados:**

das 50 amostras coletadas, foram isoladas 104 bactérias, 81,7% (85/104) gram-positivas e 18,3% (19/104) gram-negativas. Destas, 55,3% (47/85) do gênero Staphylococcus foram classificadas como multidroga resistente e resistente a meticilina e 15,7% (3/19) das gram-negativas como multidroga resistente. 66,34% (69/104) foram produtoras de biofilme, sendo 79,7% (55/69) gram-positivas e 20,3% (14/69) gram-negativas.

**Conclusões:**

tal achado traz grande preocupação visto que estes microrganismos em ambientes hospitalares apresentam risco elevado para infecções de sítio cirúrgico de difícil tratamento devido às cepas multirresistentes.

## INTRODUÇÃO

O ambiente hospitalar é um grande reservatório de microrganismos virulentos que pode acarretar aos pacientes Infecções Relacionadas à Assistência à Saúde (IRAS), principalmente em casos de internações prolongadas, procedimentos invasivos, cirúrgicos e ainda fatores exógenos relacionados ao ambiente, materiais e equipamentos. No Brasil, a preocupação com as IRAS vem crescendo desde a década de 1990, e atualmente representam a quarta causa de mortalidade em Unidade de Terapia Intensiva (UTI)^(1,2)^.

Dados da Agência Nacional de Biossegurança (ANBIO), indicam que 80% dos hospitais não controlam adequadamente as IRAS. Além disso, dados da Agência Nacional de Saúde Suplementar (ANS), informam que mais de 45 mil brasileiros morrem anualmente em decorrência destas, e para a Organização Mundial de Saúde (OMS) esse número é ainda maior, chegando a 100 mil casos por ano^(3-5)^.

A interação de diferentes microrganismos em um mesmo ambiente, principalmente hospitalar, favorece que estes desenvolvam algum mecanismo de resistência aos antimicrobianos^(6,7)^, fato este que ocorre devido a diversas mutações, dificultando o tratamento de infecções bacterianas, podendo levar a resultados clínicos desfavoráveis, e até mesmo o óbito dos pacientes^(6,8,9)^.

Outro agravante dos microrganismos hospitalares é a capacidade de produção de biofilmes, que consistem em uma camada de substância polimérica extracelular (SPE), composta por polissacarídeos, DNA extracelular, proteínas, lipídios, surfactantes, flagelos e pili, promovendo assim, proteção aos microrganismos ali presentes, sendo estes altamente resistentes a agentes antimicrobianos e desinfetantes ambientais^(10,11)^.

No interior dos biofilmes, estes microrganismos têm a capacidade de interação/comunicação entre si, processo chamado de *Quorum sensing* (QS). Este fator facilita a transferência de plasmídeos de resistência a múltiplos antimicrobianos, favorecendo o desenvolvimento de doenças infecciosas persistentes. De acordo com o Instituto Nacional de Saúde dos Estados Unidos, cerca de 80% de todas as infecções no mundo estão associadas à presença de biofilmes^(10,11)^.

Alguns ambientes hospitalares são mais suscetíveis a presença e crescimento de microrganismos, e dentre estes, encontra-se o centro cirúrgico, pois é um local de passagem de muitos pacientes e profissionais, com realização de diversos tipos de procedimentos invasivos, além de climatização artificial e ainda uso de materiais e equipamentos^(12,13)^. Estas infecções podem ser classificadas como exógenas quando advindas de fonte externa ao paciente, ou endógenas provenientes dele mesmo. A exógena tem como principal fonte de transmissão o contato direto, mas pode ocorrer de forma indireta através de equipamentos, instrumentos e superfícies contaminadas, ou ainda material de implantes processados de forma inapropriada^(14-17)^.

Devido a todos estes fatores relacionados, as IRAS, principalmente as Infecções de Sítio Cirúrgico (ISC), são uma preocupação significativa para a segurança dos pacientes, podendo ocorrer tanto em cirurgias de grande porte quanto em procedimentos menores^(18)^.

## BJETIVOS

Identificar, avaliar o perfil de resistência antimicrobiana e verificar a formação de biofilme de microrganismos isolados em superfícies de centros cirúrgicos.

## MÉTODOS

### Tipo ou delineamento de estudo

Revisão ética e aprovação foram dispensadas para este estudo, devido ao motivo de tratar-se de um estudo experimental laboratorial de coletas de amostras de superfícies, baseado no *Revised Standards for Quality Improvement Reporting Excellence* (SQUIRE 2.0)^®(19)^.

### Local

O estudo foi realizado no centro cirúrgico de duas instituições de saúde situadas no Sudoeste Goiano. Uma de média e outra alta complexidade, três e quatro salas cirúrgicas, que oferecem serviços em diversas áreas, como clínica médica, pediatria, obstetrícia, cirurgia geral, ortopedia, urologia, oftalmologia, otorrinolaringologia, entre outras.

As análises laboratoriais foram desenvolvidas no Laboratório de Bacteriologia e Micologia do curso de Biomedicina, da Universidade Federal de Jataí -GO (LBM-UFJ).

### Período e coleta das amostras

As coletas foram realizadas entre os meses de março a agosto de 2023. Para identificar, avaliar o perfil de resistência antimicrobiana e verificar a formação de biofilme foram realizadas coletas utilizando *Swabs* estéreis umedecidos em solução salina à 0,9%, friccionadas por toda extensão das superfícies e objetos selecionados, que foram: teclados e mouses de computadores, fios de aparelho aspirador, fios de bisturi elétrico, pedal do bisturi elétrico, conectores do eletrodo do monitor cardíaco e braçadeiras do monitor de pressão arterial, obtendo um total de 50 amostras.

Após a coleta os *Swabs* foram colocados em tubos de ensaio previamente identificados contendo caldo *Brain Heart Infusion* (BHI) (HIMEDIA^®^), acondicionados em caixa térmica, e transportados ao LBM-UFJ, onde permaneceram incubados por 24 h em estufa Demanda Bioquímica de Oxigênio (BOD) com temperatura de 35°C-37ºC.

### Análises microbiológicas

Pela turvação do caldo BHI (HIMEDIA^®^) foi determinado se houve ou não crescimento bacteriano. As amostras com crescimento foram semeadas por esgotamento em Ágar Sangue - AS (KASVI^®^) e incubadas por 24h em estufa BOD com temperatura de 35°C-37ºC. Após o período de incubação, foi observado se houve crescimento assim como a quantidade de colônias distintas em cada placa. As colônias diferentes morfologicamente foram re-inoculadas (isolamento secundário), para alcançar a pureza de cada uma das colônias, possibilitando a realização dos demais testes para identificação. As amostras previamente isoladas (observando padrão de hemólise e coloração de Gram) foram submetidas a provas bioquímicas tradicionais para identificação^(20)^.

### Análise de resistência e produção de biofilme

Para a realização do teste de sensibilidade aos antimicrobianos, foi utilizado o método de disco-difusão Kirby-Bauer^(21)^, depositando-se discos de antimicrobianos em placa de Petri (140x15mm) contendo ágar Mueller Hinton (HIMEDIA^®^) previamente inoculado com cepa bacteriana isolada com aproximadamente 1,5 x 10^8^ UFC/ml (0,5 da escala de *McFarland*), incubados a 35-37ºC por 24h. Os resultados foram interpretados comparando o diâmetro do halo de inibição com as classificações dos pontos de corte estabelecidos pelo *Brazilian Committee on Antimicrobial Susceptibility Testing* (BrCAST) e desta forma foram classificados como sensíveis, intermediários ou resistentes^(21)^.

Foi realizada a avaliação do perfil de resistência a oxacilina aos grupo *Staphylococcus,* (sendo os resistentes classificados como MRSA - *Staphylococcus aureus* meticilina resistente, MRSE - *Staphylococcus epidermidis* meticilina resistente e MRSS- *Staphylococcus saprophyticus* meticilina resistente), à vancomicina aos *Enterococcus* sp. (sendo os resistentes classificados como VRE - *Enterococcus* resistente à vancomicina) e as Enterobactérias ao imipenem (sendo os resistentes classificados como: possível KPC)^(21)^.

Para classificação do perfil de multirresistência, foram adotados os critérios propostos por Magiorakos *et al.* ^(22)^, onde os isolados que não apresentaram resistência ou apresentaram até duas classes foram classificados como Not MDR (*Not multi drug resistance*), os isolados que apresentaram resistência a pelo menos um representante em três ou mais classes de antimicrobiano foram classificados como MDR (*multi drug resistance*) e os isolados que apresentaram resistência a todos os agentes de todas as classes foram classificados como PDR (*pan drug resistance*)^(22)^.

Para realização dos testes de produção de biofilme, os isolados foram descongelados e uma alíquota foi transferida para tudo de ensaio contendo o caldo BHI. Após 24 horas de incubação, o biofilme foi montado em placas de 96 poços, contendo 198 μL de meio enriquecido Caldo Tríptico de Soja - TSB (HIMEDIA^®^) e 2 μL de BHI + Cepa, sendo esta incubada a 35°C-37°C em estufa BOD por 48h. Posteriormente seguiu-se as etapas descritas por Stepanovic *et al.* ^(23)^, que contemplaram a fase de leitura da placa: lavagem da placa, fixação e coloração e por fim, mensuração dos resultados em leitora de placas de microtitulação (KASVI^®^). A interpretação foi expressa através do ponto de corte *(Cut off* - DOc) descrito no Quadro 1.

**Quadro 1:** Interpretação da produção de biofilme

Classificação	Análise entre DO e DOc^*^
Não produtora de biofilme	DO ≤ DOc
Fraca produtora (+ ou 1)	DOc < DO ≤ 2X DOc
Moderada produtora (++ ou 2)	2X DOc < DO ≤ 4X DOc
Forte produtora (+++ ou 3)	4X DOc < DO

*Fonte: Stepanovic et al.^(23)^.*

## RESULTADOS

Os resultados obtidos a partir da análise microbiológica de amostras coletadas em dois hospitais apresentam uma visão abrangente da presença de microrganismos nessas instalações. Das 50 amostras analisadas, todas apresentaram crescimento de pelo menos um tipo de microrganismo, totalizando 104 bactérias isoladas. A caracterização das bactérias com base na coloração de Gram revelou uma predominância de bactérias gram-positivas, representando 81,7% (85/104) do total, em comparação com as bactérias gam-negativas, que constituíram 18,3% (19/104).

A Figura 1 fornece uma representação das espécies bacterianas identificadas (*Staphylococcus* sp*. (4), Staphylococcus epidermidis (20), Staphylococcus aureus (38), Staphylococcus saprophyticus (7), Staphylococcus coagulase negativa (11), Streptococcus viridans (1), Enterococcus* sp*. (4), Proteus mirabilis (3), Escherichia coli (7), Shigella* sp*. (3) e Acinetobacter* sp. *(5).*

**Figura 1:** Frequência de bactérias isoladas em superfícies e objetos de centros cirúrgicos de dois hospitais do sudoeste Goiano

Em relação aos mecanismos de resistência, no presente estudo foi observado que 57,7% (60/104) dos isolados apresentaram resistência aos antimicrobianos e foram classificadas como MDR de acordo com os critérios de Magioarakos *et al.^(12)^,* seguindo para avaliação da capacidade de formação de biofilme observou-se que 66,3% (69/104) dos isolados possuem esta característica, sendo 89,8% (62/69) fracas produtoras, 5,8% (4/69) moderadas produtoras e 4,4% (3/69) fortes produtoras, conforme apresentado no Quadro 2.

**Quadro 2:** Origem da amostra, perfil de resistência e capacidade de produção de biofilme

Isolado	Superfície/Objeto	Resistência as classes de antimicrobianos	Produção de Biofilme	Isolado	Superfície/Objeto	Resistência as classes de antimicrobianos	Produção de Biofilme
1	Teclado	MDR/MRSA	Não produtora	20	Fio do bisturi elétrico	MDR/MRSE	Não produtora
2	Mouse	MDR/MRSA	Não produtora	21	Pedal do bisturi	Not MDR	Não produtora
3	Mouse	MDR/MRSA	Não produtora	22	Pedal do bisturi	Not MDR	Fraca produtora
4	Teclado	MDR/MRSA	Não produtora	23	Pedal do bisturi	Not MDR	Não produtora
5	Teclado	MDR	Não produtora	24	Aparelho de PA	Not MDR	Não produtora
6	Mouse	MDR	Não produtora	25	Conector do Eletrodo	MDR/MRSE	Não produtora
7	Teclado	MDR/MRSA	Não produtora	26	Conector do Eletrodo	Not MDR	Não produtora
8	Teclado	MDR	Não produtora	27	Fio do aspirador	Not MDR	Não produtora
9	Mouse	Not MDR	Não produtora	28	Fio do aspirador	Not MDR	Não produtora
10	Teclado	MDR	Fraca produtora	29	Fio do bisturi elétrico	Not MDR	Fraca produtora
11	Mouse	MDR/MRSA	Não produtora	30	Fio do bisturi elétrico	MDR/MRSE	Fraca produtora
12	Braçadeira de PA	Not MDR	Não produtora	31	Fio do bisturi elétrico	MDR	Não produtora
13	Braçadeira de PA	Not MDR	Fraca produtora	32	Pedal do bisturi elétrico	Not MDR	Não produtora
14	Braçadeira de PA	MDR/MRSE	Média produtora	33	Pedal bisturi	MDR	Fraca produtora
15	Conector do Eletrodo	MDR/MRSA	Fraca produtora	34	Pedal bisturi	MDR/MRSE	Fraca produtora
16	Conector do Eletrodo	MDR/MRSE	Não produtora	35	Braçadeira de PA	MDR/MRSA	Fraca produtora
17	Conector do Eletrodo	MDR/MRSA	Não produtora	36	Braçadeira de PA	MDR/MRSE	Não produtora
18	Fio do aspirador	Not MDR	Fraca produtora	37	Conector do Eletrodo	MDR/MRSE	Fraca produtora
19	Fio do aspirador	Not MDR	Fraca produtora	38	Fio do aspirador	Not MDR	Fraca produtora
39	Fio do aspirador	Not MDR	Fraca produtora	60	Teclado 3	Not MDR	Não produtora
40	Fio bisturi elétrico	Not MDR	Fraca produtora	61	Teclado 3	Not MDR	Fraca produtora
41	Fio bisturi elétrico	MDR	Fraca produtora	62	Teclado 3	MDR/MRSA	Fraca produtora
42	Pedal bisturi	Not MDR	Não produtora	63	Mouse 3	MDR	Forte produtora
43	Pedal bisturi	Not MDR	Fraca produtora	64	Mouse 3	Not MDR	Fraca produtora
44	Teclado 1	MDR	Fraca produtora	65	Teclado 4	Not MDR	Moderada produtora
45	Teclado 1	Not MDR	Fraca produtora	66	Teclado 4	MDR/MRSA	Fraca produtora
46	Teclado 1	Not MDR	Fraca produtora	67	Mouse 4	MDR	Fraca produtora
47	Teclado 2	Not MDR	Fraca produtora	68	Teclado 5	MDR	Fraca produtora
48	Teclado 2	Not MDR	Não produtora	69	Mouse 5	MDR	Moderada produtora
49	Teclado 2	MDR/MRSE	Fraca produtora	70	Teclado 6	MDR/MRSA	Fraca produtora
50	Teclado 2	MDR/MRSA	Fraca produtora	71	Mouse 6	MDR/MRSA	Fraca produtora
51	Mouse 1	MDR/MRSA	Fraca produtora	72	Mouse 6	MDR/MRSS	Fraca produtora
52	Mouse 1	Not MDR	Não produtora	73	Aparelho de PA	MDR/MRSS	Fraca produtora
53	Mouse 1	Not MDR	Não produtora	74	Aparelho de PA	MDR/MRSA	Não produtora
54	Mouse 2	Not MDR	Não produtora	75	Aparelho de PA	MDR/KPC	Fraca produtora
55	Mouse 2	MDR	Fraca produtora	76	Aparelho de PA	Not MDR	Fraca produtora
56	Mouse 3	MDR	Não produtora	77	Conector do Eletrodo	MDR/MRSE	Fraca produtora
57	Mouse 4	Not MDR	Fraca produtora	78	Conector do Eletrodo	MDR/KPC	Fraca produtora
58	Mouse 5	MDR/MRSE	Fraca produtora	79	Fio aspirador	Not MDR	Não produtora
59	Teclado 3	MDR/MRSE	Fraca produtora	80	Fio bisturi elétrico	Not MDR	Não produtora
81	Fio bisturi elétrico	Not MDR	Fraca produtora	93	Fio aspirador	MDR/MRSA	Fraca produtora
82	Pedal bisturi	MDR/MRSS	Não produtora	94	Fio bisturi elétrico	MDR/MRSA	Fraca produtora
83	Aparelho de PA	Not MDR	Fraca produtora	95	Fio bisturi elétrico	Not MDR	Fraca produtora
84	Aparelho de PA	Not MDR	Moderada produtora	96	Pedal bisturi	MDR/MRSA	Fraca produtora
85	Conector do Eletrodo	MDR/MRSA	Fraca produtora	97	Pedal bisturi	Not MDR	Fraca produtora
86	Fio aspirador	MDR/MRSS	Fraca produtora	98	Pedal bisturi	Not MDR	Fraca produtora
87	Fio bisturi elétrico	MDR/MRSS	Fraca produtora	99	Pedal bisturi	Not MDR	Fraca produtora
88	Pedal bisturi	MDR/MRSS	Forte produtora	100	Pedal bisturi	MDR/MRSA	Fraca produtora
89	Pedal bisturi	Not MDR	Fraca produtora	101	Fio do monitor	MDR	Fraca produtora
90	Aparelho de PA	MDR/MRSA	Fraca produtora	102	Fio do monitor	Not MDR	Fraca produtora
91	Aparelho de PA	MDR/MRSA	Fraca produtora	103	Fio do monitor	MDR/MRSE	Fraca produtora
92	Conector do Eletrodo	MDR/MRSA	Forte produtora	104	Pedal do bisturi	Not MDR	Fraca produtora

*Not MDR - Not drug resistance; MDR - multi drug resistance; MRSA - Staphylococcus aureus meticilina resistente; MRSE - Staphylococcus epidermidis meticilina resistente; MRSS - Staphylococcus saprophyticus meticilina resistente; PA - pressão arterial; KPC - grupo klebsiella produtora de carbapenemase.*

## DISCUSSÃO

Dentre a IRAS a ISC se destaca por apresentar grande preocupação no ambiente hospitalar, e podem ser causadas por vasta gama de bactérias sejam gram-negativas e/ou gram-positivas^(17)^. No centro cirúrgico, quando as medidas de prevenção não são implantadas ou ainda negligenciadas, o risco de uma infecção aumenta consideravelmente, podendo gerar danos temporários ou permanentes, e até mesmo o óbito dos pacientes^(24)^.

No presente estudo evidenciou-se que em todas as superfícies nas quais houve coleta, foram isolados microrganismos, e dentre estas foram encontradas espécies causadoras de IRAS, com significativo perfil de resistência aos antimicrobianos, além da capacidade da produção de biofilme. Estes achados corroboram com estudo realizado em um hospital de Belo Horizonte, como o mesmo objetivo, que aponta dados significativos relacionados à incidência de microrganismos em superfícies e objetos de um centro cirúrgico, sendo estes: *Acinetobacter baumannii, Enterobacter* sp. e *Staphylococcus* coagulase negativa, bactérias possíveis causadores de IRAS destacando ISC, tendo em vista a presença de isolados com alto perfil de resistência aos antimicrobianos^(25)^.

Outro estudo^(13)^, avaliou o perfil bacteriano encontrado nas superfícies e equipamentos de um bloco cirúrgico, e em todas estas foram isolados microrganismos, com prevalência de cocos gram-positivos, sendo *S. aureus* o mais incidente, corroborando com os achados deste estudo, onde todas as superfícies e objetos testados apresentaram crescimento bacteriano, com predomínio do gênero *Staphylococcus,* destacando a prevalência de *S. aureus.* Este patógeno compõe a microbiota da pele humana, mas pode causar diversas infecções em ambiente hospitalar, tais como infecções respiratórias, sistêmicas e de sítio cirúrgico.

Pesquisa similar^(26)^ também apontou maior prevalência da espécie *S. aureus,* a qual está intimamente ligada ao desenvolvimento IRAS principalmente por cepas resistentes a meticilina (MRSA). No presente estudo 77,8% (81/104) dos isolados são do gênero *Staphylococcus,* destes 58,02% (47/81) apresentaram resistência a oxaciclina classificados como MDR/MRSA, e 66,66% (54/81) são cepas produtoras de biofilme. Vale ressaltar que esta espécie possui a capacidade de produzir biofilme o que dificulta a resposta imunológica e terapêutica farmacológica^(17)^.

*Enterococcus* sp. compreende um gênero de bactérias gram-positivas naturalmente encontradas no trato gastrointestinal, no entanto estudos apontam a presença de espécies de gênero com infecções hospitalares, apresentando relevância quando estas desenvolvem mecanismos de resistência contra vancomicina (VRE), visto que podem provocar infecções oportunistas e de fácil propagação no ambiente hospitalar. Estudo realizado em um hospital universitário^(12)^, aponta elevados índices de prevalência por *Enterococcus* sp. em diversos setores do hospital, sendo o segundo mais encontrado nas amostras de secreção de incisões cirúrgicas. Este estudo por sua vez isolou 3,84% (05/104) amostras, e destas todas se mostraram sensíveis à vancomicina, no entanto, 75% (03/04) dos isolados foram classificados como MDR, apontando assim risco eminente de desenvolvimento de ISC, tendo em vista que estes microrganismos são produtores de polissacarídeo de superfícies (*antígeno polissacarídeo enterocócico*) essencial para o desenvolvimento de biofilme, o qual está diretamente ligado com a resistência aos antimicrobianos bem como na patogênese^(27)^.

No presente estudo 18,3% (19/104) das bactérias isoladas foram espécies gram-negativas (*E.coli, Proteus mirabilis, Acinetobacter sp. e Shigella sp.),* onde 21,05% (04/19) apresentaram multirresistência (MDR) aos antimicrobianos testados. Ademais, este estudo também corrobora com os achados de uma pesquisa^(28)^ realizada que aponta que as superbactérias são um dos maiores desafios enfrentados no ambiente hospitalar principalmente nas ISC.

Além disso, a capacidade de produção de biofilme foi avaliada, sendo uma característica associada à virulência e resistência bacteriana. A presença de biofilme pode contribuir para a persistência das IRAS e a dificuldade no tratamento adequado^(29)^. A análise revelou variações na produção de biofilme, 66,34% (69/104) dos isolados foram classificados produtores de biofilme, 79,7% (55/69) em bactérias gram-positivas e 20,3% (14/69) em gram-negativas. Assim como um estudo^(30)^ realizado em um hospital de Pokhara no Nepal, que avaliou a contaminação de objetos altamente tocáveis, e nas 181 amostras analisadas, verificou maior prevalência de *S. aureus* sendo que 36,3% foram resistentes a meticilina e 31,8% produtores de biofilme.

### Limitações do estudo

Ao passo em que ocorria a identificação das cepas bacterianas, era necessário realizar vários repiques de uma mesma amostra para que a colônia fosse isolada para prosseguir com o fluxograma de identificação. Com isso, em uma única amostra era necessário em torno de 5 a 6 dias para ser identificada, devido ao fato de uma única placa apresentar crescimento polimicrobiano.

### Contribuições para a área da enfermagem, saúde ou política pública

As IRAS podem ser prevenidas e controladas através da implementação de medidas de prevenção e controle de infecções em todos os níveis de cuidados de saúde. A prevenção das IRAS é responsabilidade das instituições de saúde e de toda a equipe de colaboradores. Todos devem trabalhar juntos para reduzir o risco de infecção tanto para os pacientes como para os próprios trabalhadores da saúde^(31)^. A associação entre a resistência antimicrobiana e a produção de biofilme destaca a necessidade de implantação de estratégias integradas de controle de infecções, ressaltando o papel fundamental dos profissionais de saúde, a identificação precoce das bactérias e a implementação de medidas preventivas adequadas fundamentais para mitigar os riscos de infecções hospitalares associadas a esses microrganismos^(32,33)^.

## CONCLUSÕES

Os resultados apresentados fornecem *insights* valiosos para a gestão de saúde hospitalar, destacando a importância da vigilância contínua, práticas de controle de infecções e o desenvolvimento de estratégias terapêuticas personalizadas para abordar a diversidade microbiológica e a resistência antimicrobiana em ambientes hospitalares. Diante de tais achados, torna-se necessário que os colaboradores que atuam no Centro Cirúrgico (CC), adotem melhores medidas de limpeza e desinfecção das superfícies e objetos a fim de diminuir o risco de infecções cruzadas.

## Data Availability

Os dados de pesquisa estão disponíveis no corpo do artigo.
